# The Missing Half of Language Learning in Current Developmental Language Models: Exogenous and Endogenous Linguistic Input

**DOI:** 10.1162/OPMI.a.33

**Published:** 2025-09-17

**Authors:** Nan Zhao, Xufeng Duan, Zhenguang G. Cai

**Affiliations:** Department of Translation, Interpreting and Intercultural Studies, Hong Kong Baptist University, Hong Kong SAR; Department of Linguistics and Modern Languages, The Chinese University of Hong Kong, Hong Kong SAR; Brain and Mind Institute, The Chinese University of Hong Kong, Hong Kong SAR

**Keywords:** developmental language models, language learning, label feedback, private speech, inner speech, sleep consolidation

## Abstract

Developmental language models (DLMs) aim to replicate the efficiency of child language acquisition but often focus solely on the estimation of exogenous linguistic input. We argue that a child’s linguistic growth is also critically shaped by endogenous processes, including (1) co-opting language in non-linguistic perception and cognition, (2) engaging in private and inner speech, and (3) benefiting from neural replay of linguistic information during sleep. These endogenous processes amplify and refine exogenous linguistic input in ways that current DLMs do not replicate. To align DLMs with child language acquisition, we propose redefining “linguistic exposure“ to encompass both exogenous and endogenous linguistic input. By integrating label feedback, self-generated speech, and sleep-like consolidation, researchers can narrow the gap between artificial and human learning. Collaborations across machine learning, psychology, and linguistics will be essential to ground models in empirical data on child behavior and build DLMs that truly reflect the marvel of language acquisition.

## LINGUISTIC INPUT IN CHILDREN AND DEVELOPMENTAL LANGUAGE MODELS

As transformer-based language models (LMs), such as the GPT series, have demonstrated remarkable abilities in understanding and producing human language (e.g., Brown et al., 2020; Cai et al., 2024), the pursuit of cognitively plausible *developmental* language models (DLMs) has emerged as a critical frontier in computational linguistics and cognitive science (Baroni, 2022; Dupoux, 2018; Linzen, 2019). DLMs seek to replicate the efficiency of child language acquisition, which occurs robustly despite limited exogenous linguistic input—a phenomenon starkly at odds with the data-hungry nature of modern artificial intelligence systems (McClelland et al., 2020; Warstadt & Bowman, 2022). By bridging insights from linguistics, developmental psychology, and neuroscience, such models hold promise for elucidating the mechanisms underlying human language learning while advancing AI systems that generalize from sparse data (Lake et al., 2017; Linzen, 2020).

However, the validity of LMs and DLMs as models of human cognition remains contentious (Futrell & Mahowald, 2025; Shah & Varma, 2025). Critics highlight fundamental divergences: children learn language through embodied, socially embedded interactions (Tomasello, 2005), guided by innate biases (e.g., hierarchical structure detection; Bloom, 2000) and real-time feedback (Smith & Yu, 2008), whereas LMs and DLMs depend on statistical patterns in static text corpora (Chemero, 2023). Crucially, human language acquisition is intertwined with world knowledge—children ground words in sensorimotor experiences and goal-directed play (Lake et al., 2017)—a process absent in current LM and DLM architectures. While these qualitative mismatches are significant, in this opinion piece, we focus not on these qualitative mismatches but on a quantitative concern: the *scale* of training data used in DLM research.

A common assumption in the field is that cognitively plausible DLMs should be trained on data similar in quantity to what children are exposed to during their language learning process (van Schijndel et al., 2019). A prominent example is the BabyLM Challenge, a community initiative that pushes models to learn from roughly the amount of exogenous linguistic data available to a child. Specifically, the BabyLM shared task restricts training to 10 million or 100 million words drawn from child-directed and child-relevant sources. The goal is to discover how an LM can reach competent language understanding under human-like input limitations (Warstadt, Choshen, et al., 2023; Warstadt, Mueller, et al., 2023). This data-efficiency assumption stems from the observation that children master language with relatively limited explicit linguistic input compared to the vast datasets used to train state-of-the-art LMs. For instance, Gilkerson et al. (2017) estimated that children are exposed to approximately 2–7 million words per year, amounting to around 100 million words by age 12. In contrast, current LMs are typically trained on billions or even trillions of words (Brown et al., 2020; Raffel et al., 2020).

However, estimating training data based on *exogenous* linguistic input alone fails to account for the *endogenous* linguistic exposure children experience. We argue that a child’s linguistic growth is not merely a product of exogenous input but is critically shaped by endogenous processes including (1) linguistic labelling during perception and cognition (e.g., automatically activating the label *train* when seeing a toy train), (2) inner and private speech (e.g., covertly generating *the train moves*), and (3) neural replay of linguistic information during sleep. These endogenous processes amplify and refine exogenous input in ways that current DLMs do not replicate.

## IMPLICITLY CO-OPTING LANGUAGE IN NON-LINGUISTIC PERCEPTION AND COGNITION

Language is deeply interwoven with perception and cognition, creating feedback loops that augment linguistic input (Lupyan & Bergen, 2016; Lupyan et al., 2020). For children, encountering an object like a ball triggers automatic activation of its label “ball”, even without external verbal input—a phenomenon termed *label feedback* (Lupyan, 2012). Experimental work demonstrates that infants as young as 18 months exhibit priming effects when images are paired with labels (Mani & Plunkett, 2010), indicating preverbal activation of linguistic representations.

Label feedback plays a crucial role in vocabulary acquisition and semantic network development, with the automatic activation of labels strengthening bidirectional connections between object knowledge and linguistic representations. Children shown novel objects with labels form sharper conceptual boundaries than those without (Dessalegn & Landau, 2008; Ferry et al., 2010). After learning shape-based categories through labelled exemplars, children also showed enhanced attention to shape when learning new object names, even in the absence of explicit shape cues (Yoshida & Smith, 2005). Linguistic labels are also a powerful tool for categorization, fostering cultural stability, enabling conceptual innovation, and guiding the development of category knowledge from a young age (Gelman & Roberts, 2017). These findings indicate that label feedback is not merely a general enhancement mechanism but rather shapes language development in grammatically specific ways, ultimately scaffolding children’s ability to use language as a tool for organizing and reasoning about their perceptual experiences.

Thus, linguistic knowledge is not merely acquired through passive input but is actively recruited to parse the world. This creates a self-reinforcing cycle: labels refine perception, which in turn deepens linguistic understanding (Mani & Ackermann, 2018). Currently, LMs and DLMs trained only on explicit text lack this bidirectional interface between language and cognition, omitting a key driver of children’s data efficiency.

## PRIVATE AND INNER SPEECH IN CHILDREN

Children’s linguistic growth is further propelled by *private speech* (audible self-talk) and *inner speech* (covert verbal thought), which internalize social dialogue into tools for self-regulation (Vygotsky, 2012). Private speech enables rehearsal of vocabulary and syntax (e.g., narrating play: “The train goes choo-choo!”), while inner speech supports silent planning and problem-solving (Alderson-Day & Fernyhough, 2015). Correlational studies have documented associations between these behaviours and language development: preschoolers who use frequent private speech tend to show stronger vocabulary growth (Winsler et al., 2003), while language-delayed children often exhibit less mature self-talk (Winsler et al., 2007), though the causal direction of these relationships remains to be established.

Private speech appears to be associated with enhanced cognitive control. In executive function tasks, children who verbalize instructions (“I need to sort by color, then size”) typically perform better than silent peers (Fernyhough & Fradley, 2005). Longitudinal observations indicate that private speech becomes increasingly internalized and task-focused with age, coinciding with advances in self-regulation (Winsler et al., 2000). This self-directed linguistic practice effectively doubles children’s exposure: they learn from both caregiver input and their own verbal experimentation.

Unlike children, who test hypotheses (“Is it *runned* or *ran*?”) and refine their knowledge through error (e.g., overgeneralization and correction), prevalent LMs and DLMs lack mechanisms for generating or learning from self-produced language and passively absorb preexisting text. This absence of metacognitive verbal rehearsal represents a critical gap in developmental plausibility.

## NEURAL REPLAY OF LINGUISTIC INFORMATION DURING SLEEP

Sleep plays a vital role in cognitive development and learning, particularly in memory consolidation in language learning. The brain has a routine mechanism of *neural replay* during sleep (Wilson & McNaughton, 1994), reactivating and replaying neural patterns associated with experiences (e.g., language communication and learning) from the waking state. This replay is not a mere passive recapitulation of daily events but an active process that strengthens neural connections and facilitates the integration of new information into existing knowledge networks (Rasch & Born, 2013; Spens & Burgess, 2024). Thus, when children sleep, their brains revisit and reprocess the language input they’ve been exposed to during the day, including words, phrases, grammatical structures, and even subtle phonetic distinctions.

The replay of linguistic experiences during sleep serves several important functions, such as memory consolidation, pattern extraction, integration with existing knowledge, and generalization (Davis & Gaskell, 2009). For instance, Dumay and Gaskell (2007) demonstrated that newly learned words only began to compete with existing words in lexical decision tasks after a period of sleep, suggesting that sleep is necessary for new words to be fully integrated into the mental lexicon. Similarly, Gómez et al. (2006) showed that infants were better able to extract the underlying grammatical structure from artificial languages after a period of sleep. In the domain of phonological learning, Fenn et al. (2003) found that adults’ ability to recognize synthetic speech improved significantly after a night’s sleep, but not after an equivalent period of wakefulness, suggesting that sleep consolidates phonological learning. Tham et al. (2015) demonstrated that the semantic integration of newly learned words, as measured by semantic priming effects, was enhanced after sleep. Moreover, Henderson et al. (2015) showed that children who napped after learning new words showed better long-term retention of those words compared to children who did not nap.

These processes enable children to build robust, generalized linguistic knowledge from sparse data. In contrast, LMs and DLMs undergo no analogous consolidation phase; their “knowledge” is fixed post-training, lacking the dynamic refinement seen in humans. Integrating sleep-like mechanisms—such as offline memory reprocessing—could help DLMs achieve similar efficiency.

## SIMULATING ENDOGENOUS PROCESSES OF CHILD LANGUAGE LEARNING IN DEVELOPMENTAL LANGUAGE MODELLING

DLMs could simulate language co-activation by incorporating multi-modal or context-driven token activations. One measure is effective vocabulary activation size—counting unique tokens a model internally associates with non-linguistic contexts. In multi-modal LMs, we could measure how many words the model predicts when given an image or sound, similar to a child implicitly labelling what they see. High co-activation would manifest as a broader spread of tokens being internally triggered beyond what appears in external input. Attention weight shifts serve as another analogue: tracking changes in an LM’s attention patterns when non-linguistic context is present. Recent multimodal transformer models already hint at this alignment of visual and textual representations (Tsai et al., 2019; Wang et al., 2024). By introducing perceptual contexts and measuring vocabulary activation breadth or attention shifts, we simulate implicit labelling, acknowledging that a child’s effective exposure includes moments when perception invokes language even without external speech.

To incorporate private and inner speech analogues, models can generate and consume their own pseudo-data during training. One approach implements a “self-dialogue” phase: having the model produce a sequence (a thought or explanation) given a context, then feeding this back as additional input, simulating an internal conversation similar to chain-of-thought prompting (Wei et al., 2022). The hidden-state divergence between normal inference and inference with a self-generated “inner monologue” could measure internal speech activation. Attention weight shifts during “thinking aloud” might indicate the model retrieving knowledge differently, similar to how inner speech draws on disparate memories. Group Relative Policy Optimization (GRPO), introduced in DeepSeek-R1 (Guo et al., 2025), offers a promising framework by allowing a model to generate multiple candidate outputs, evaluate them against each other, and reinforce higher-quality generations without requiring a learned reward model. This simulates self-assessment, producing and selecting among utterances based on utility—similar to how children verbally test hypotheses. Operationally, we could insert unlabelled segments where the model generates plausible next text and then continues, training on its own output. GRPO provides a structured framework for evaluating these self-generated utterances, conceptually aligned with how children refine linguistic hypotheses through private or inner speech and receive reinforcement when successful forms are later used in interaction (Winsler et al., 1997). Success would be measured by improvements in downstream performance, analogous to benefits seen in children who use self-talk.

Sleep-associated replay can be emulated through offline experience replay (Kumaran et al., 2016). After training on a mini-batch (wake experience), a “sleep phase” could have the model generate pseudo-examples or replay portions of training data with variation. Recent work by Muennighoff et al. (2023) indicates that reusing textual data up to four times in different orders does not degrade performance and may even lead to modest improvements. This suggests that, in data-constrained settings, repeated use of training data can be a practical and effective strategy. For a DLM with limited input tokens, multiple shuffled passes through the data could simulate a child’s nights of sleep. “Replay gradients” could measure how much the model changes due to replay alone. Inspiration can also come from generative replay methods in continual learning (Rolnick et al., 2019). LAMOL (Sun et al., 2019) trains models to replay previous tasks by generating pseudo-samples to avoid forgetting. Similarly, DLMs could periodically generate continuations of earlier texts for training, simulating “dreaming” consistent with past experiences. Tadros et al. (2022) implemented a sleep-inspired phase in neural networks and found that “spontaneous replay simulating sleep-like dynamics” prevented catastrophic forgetting. For DLMs, an unsupervised rehearsal stage could feed contexts from prior training with a lower learning rate. Measuring impact could involve comparing representation stability before vs. after replay or tracking perplexity on earlier data. These approaches provide insight into effective exposure from internal processes. If a DLM needs multiple replay passes to mimic human retention, this might correspond to a child replaying linguistic experiences over several nights. Quantifying gradient updates or pseudo-text needed helps estimate the amplification factor that internal processes contribute to learning. A child’s 100 million external tokens might effectively become 300 million tokens after accounting for replay—suggesting why human children achieve so much from comparatively little data. Incorporating these mechanisms uses time in lieu of extra data, much as evolution has enabled humans to enhance learning during offline hours.

While simulating these endogenous mechanisms introduces additional computational steps—such as generating pseudo-data, replaying previous experiences, or analyzing multimodal co-activation—this added complexity could be offset by gains in data efficiency. Unlike scaling via ever-larger datasets, these processes leverage more intelligent use of limited input, affording more developmental plausibility in the modelling of language learning. Moreover, these mechanisms need not be applied continuously; they could be selectively engaged during training epochs (e.g., interleaved replay phases or occasional self-dialogue rounds), striking a balance between computational demands and learning benefits. Future small-scale pilot studies could help quantify these trade-offs more precisely, shedding light on when and how these mechanisms yield the most value relative to their cost.

## CONCLUSION

In conclusion, developmental language modelling should move beyond the simplistic metric of input token counts and toward a more holistic accounting of linguistic exposure. By delineating the endogenous processes in language learning—perceptual co-activation, private/inner speech, and sleep replay—we acknowledge that a child’s brain enriches and multiplies the raw language it encounters. Each process contributes a different facet: co-activation ties language to multisensory events (providing embedded, contextualized exposures), private speech generates new utterances and reinforces self-regulation (providing self-supervised practice), and neural replay consolidates and abstracts knowledge (providing retention and generalization of exposures). We have offered suggestions regarding how these endogenous processes could be operationalized in DLMs through concrete technical proxies, all informed by recent findings in cognitive science and machine learning.
